# Vaccine Development against COVID-19: Study from Pre-Clinical Phases to Clinical Trials and Global Use

**DOI:** 10.3390/vaccines9080836

**Published:** 2021-07-29

**Authors:** Sagheer Ahmed, Saeed Khan, Imran Imran, Fadwa Al Mughairbi, Fahad Sultan Sheikh, Javid Hussain, Ajmal Khan, Ahmed Al-Harrasi

**Affiliations:** 1 Department of Basic Medical Sciences, Shifa College of Pharmaceutical Sciences, Shifa Tameer-e-Millat University, Islamabad 44000, Pakistan; sagheer.scps@stmu.edu.pk (S.A.); fssheikh1999@gmail.com (F.S.S.); 2 Dow International Medical College, Dow University of Health Sciences, Karachi 74200, Pakistan; saeed.khan@duhs.edu.pk; 3 Department of Pharmacology, Faculty of Pharmacy, Bahauddin Zakariya University, Multan 60800, Pakistan; imran.ch@bzu.edu.pk; 4 Department of Clinical Psychology, College of Medicine & Health Sciences, UAE University, Al Ain 15551, United Arab Emirates; f.almughairbi@uaeu.ac.ae; 5 Department of Biological Sciences and Chemistry, University of Nizwa, P.O. Box 33, Birkat Al Mauz, Nizwa 616, Oman; javidhej@unizwa.edu.om; 6 Natural and Medical Sciences Research Center, University of Nizwa, P.O. Box 33, Birkat Al Mauz, Nizwa 616, Oman

**Keywords:** COVID-19, vaccine, pharmaceutical companies, global demand

## Abstract

As per the World Health Organization (WHO), more than 288 vaccines against COVID-19 are being developed, with an estimated 184 being presently investigated in the pre-clinical phases, while 104 of these vaccine candidates are at various stages of clinical trials. Twelve of these are in the advanced stages of clinical investigation, and promising results in the phase 3 trials have already paved the way for their regulatory approval and subsequent dissemination for global use. Preliminary and interim results of some of these candidate vaccines are being analyzed for public dissemination. Some of these vaccines have already been rolled out to immunize not only the highest risk individuals but also the general population in several countries. Once their safety and efficacy are established, the next limiting step would be their mass manufacturing by the pharmaceutical companies to fulfill the global demand. The challenge of manufacturing billions of doses of high-quality vaccines is under-appreciated at the moment. A massive vaccination drive would be needed to protect people of all ages. The timely and coordinated execution of the vaccination effort would require unprecedented coordination at the national and international levels for generating funds to purchase the required doses of vaccines, fair distribution of doses and managing the mechanics of delivering vaccines throughout the world.

## 1. Introduction

**Figure 1:**
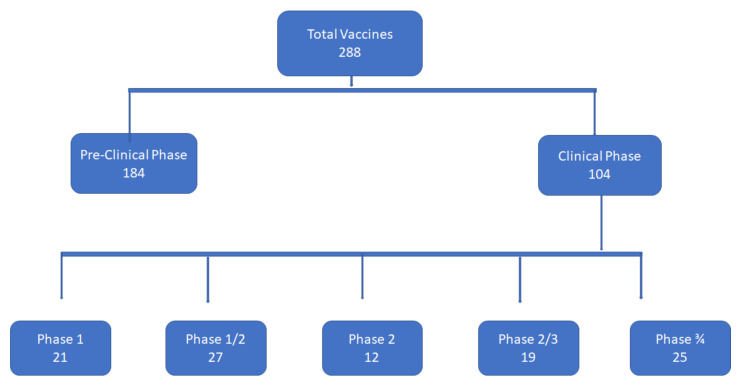
Vaccine development against COVID-19; number of vaccines in pre-clinical and clinical phases, in phase I, phase I/II, phase II, phase II/III, and phase III.

With the coronavirus disease 2019 (COVID-19) pandemic becoming a global crisis, organizations from all across the globe have come together to lead the fight against the disease. As a result, concerted efforts to develop both a safe and efficacious vaccine are taking place. However, the final decision to approve a vaccine must be based on the risk/benefit calculations grounded in sound science [1]. More than two hundred different vaccines are being investigated for their potential role in stemming the COVID-19 pandemic [2] (Figure 1). Currently, only a handful of vaccines are approved based on the preliminary data obtained from their ongoing phase III clinical trials. Therefore, finding safer and more effective vaccines to protect the mass populace from contracting the severe acute respiratory syndrome coronavirus 2 (SARS-COV-2-CoV-2) is paramount. Vaccines are therapeutic agents which, when administered, initiate an adaptive immune response against the foreign agent, whether bacterial, viral or otherwise, henceforth protecting the host from later infections by the respective pathogens. Multiple approaches are used to induce immunity against the pathogens, and the resulting vaccines, especially those designed to protect against measles, pertussis, tetanus diphtheria and influenza, save countless lives every year. Now, the lessons learnt from the past are being employed at a pace never seen before to develop safe, effective and inexpensive vaccines against the novel SARS-CoV-2. Several different companies developing these vaccines are using many different platforms and strategies, as summarized in Table 1, to synthesize vaccines to battle the pandemic.

SARS-CoV-2, belonging to the family *Coronaviridae*, has been harmful to both human and animal health in the past. At the beginning of the millennium, severe acute respiratory syndrome coronavirus (SARS-CoV) was responsible for infecting several thousand people [3,4]. Since 2012, Middle East respiratory syndrome coronavirus (MERS-CoV) has infected about a couple of thousand individuals, but its fatality rate was much higher than SARS-CoV [5,6]. Now SARS-CoV-2 is wreaking havoc throughout the world.

The antigenic forms of the spike (S) protein, which is widely thought to be the primary target of protective immunity, are being employed in most of the COVID-19 vaccines [7,8,9,10]. The S protein is the main target of neutralizing antibodies and is responsible for binding to angiotensin-converting enzyme 2 (ACE), after which the virus gains entry into the cell [11,12,13]. Animal studies have shown that this approach is successful in producing neutralizing antibodies, especially since it was found that introduction of the S antigen in rhesus macaques afforded protective immunity [14]. This suggests that protective immunity can be produced by using the S antigen to elicit an immune response. Other studies suggest that even without producing the neutralizing antibodies, the immune response initiated by the S protein may be good enough to combat SARS-CoV-2 infection through cellular immunity [7,15,16].

## 2. Vaccine for COVID-19 by the United Kingdom

### University of Oxford/AstraZeneca, UK

The vaccine developed by the pharmaceutical firm AstraZeneca in Cambridge, UK and the University of Oxford, UK, was anticipated eagerly because unlike vaccines from Pfizer and BioNTech, which require storage at very low temperatures, the Oxford/AstraZeneca vaccine is relatively easy to distribute and store, although the storage temperature for the Pfizer and BioNTech vaccine since been has been relaxed to −25 to −20 °C for two weeks. The results of their phase 3 clinical trial, although initially disseminated through press releases, were the first phase 3 results published for a vaccine among the leading COVID vaccines. These results were subsequently peer-reviewed and published in The Lancet journal [17]. The published data were scrutinized heavily, in part because this vaccine could be the cheapest of the currently developed leading vaccines and in part due to the concerns about its safety in the elderly and determining the most effective dose for providing optimal protection against the disease.

Developed by a partnership between AstraZeneca and the University of Oxford, the vaccine was initially called AZD1222. It was synthesized by AstraZeneca and then entered human trials for further investigation. The formulation in essence is a viral vector vaccine which lacks the ability to replicate after entering the cells of the host. It contains the S protein, which is the primary immunogenic protein [18]. The safety and immunogenic profiles of the vaccine have already been reported in early phase clinical trials [19].

Their phase III study was named “A Phase III Randomized, Double-blind, and Placebo-controlled Multicenter Study in Adults to Determine the Safety, Efficacy, and Immunogenicity of AZD1222, a Non-replicating ChAdOx1 Vector Vaccine, for the Prevention of COVID-19” [20,21]. This investigation started in August 2020 and the interim results were known by December 2020. Around 30,000 participants were enlisted. The vaccine comprises two dosages (Table 2), separated by about a month, given through the intra-muscular route. The primary outcome of the investigation was finding the protection afforded by the vaccine 14 days after the second dose of the vaccine. Secondary outcomes included antibody levels one year after the second dose administration.

Preliminary data released by the group previously suggested that a subset of participants who were accidently administered a lower first dose of the vaccine exhibited higher protection rate. This unexpected finding increased the curiosity of the scientific community, as it was difficult to explain it. As the data from the different studies were compiled rather than obtained from a single clinical trial, some scientists expressed concern that the data should not have been pooled together. The results obtained for the standard regimen show an efficacy of 62% at preventing the symptomatic disease, while the regimen which includes a lower initial dose displayed an efficacy of 90%. However, combining the data from various dosing regimens yielded an efficacy of 70%.

The trial investigators responded to the pooling of the data, saying it was done as per guidance from the regulators. However, they could not explain how the efficacy of a lower dose was higher than that of a higher dose of the vaccine and hinted at a separate trial to follow up on this finding. Another point of concern was that no elderly participants were found in the low-dose regimen group, suggesting that the gain in the efficacy might be a result of excluding the elderly who historically have a low immune response to vaccines but are more vulnerable to the disease.

However, one investigator of the study pointed out that further analysis of their data, which they carried out at the request of *The Lancet* reviewers, suggests that the efficacy of the low-dose groups was higher in all age groups under 55. Since only 12% participants were above the age of 55, it is not clear how much this group would have benefited from even the standard dose of the vaccine. However, previous studies suggest that in the older adults whose immune response was comparable to the younger participants, the vaccine is likely to work well [22].

## 3. Vaccines for COVID-19 by the United States

### 3.1. Moderna/NIAID, USA

Recently published results of a clinical trial [23] show that an RNA-based vaccine can provide a protective immune response against SARS-CoV-2 when measured two weeks post-first injection. The vaccine was recently approved by US regulators. Moderna, Inc., a biotechnology company based in the United States that specializes in clinical stage bio-technology, is sponsoring and just completed an advanced phase III human study of this vaccine called mRNA-1273 against COVID-19. Within animal models, the vaccine has successfully induced protective immunity against the virus [24]. The primary purpose of the trial was to evaluate the efficacy, safety, and immunogenicity of mRNA-1273 to prevent SARS-CoV-2 infection up to 2 years after the second dose of the investigational vaccine. This clinical trial, which enrolled approximately 30,000 participants, was officially named “A Phase 3, Randomized, Stratified, Observer-Blind, Placebo-Controlled Study to Evaluate the Efficacy, Safety, and Immunogenicity of mRNA-1273 SARS-CoV-2 Vaccine in Adults Aged 18 Years and Older”. It was started on 27 July 2020. The lead, mRNA-1273, comprises nucleoside-altered, lipid nanoparticle–encased courier RNA (mRNA) and a SARS-CoV-2 spike (S) glycoprotein in its prefusion state. This S glycoprotein is principal for viral infiltration into the host cell by moderating virus–cell interactions. The randomized, placebo-controlled preliminary study took place in the United States, where participants were administered with one intramuscular (IM) injection of 100 micrograms (ug) of mRNA-1273 on Day 1 and on Day 29 in the trial arm and 0.9% sodium chloride infusion in the placebo comparator arm [25]. The primary outcome was protection from SARS-CoV-2. Secondary outcomes included prevention from the extreme COVID-19 ailment (as assessed by the duration of hospital stay and oxygen requirements) and preventing asymptomatic infections.

As early as January 2020, Moderna announced that they had a promising vaccine candidate against SARS-CoV-2, termed as mRNA-1273. The first phase of human investigations of the vaccine candidate started in March 2020, in association with the US National Institute of Allergy and Infectious Diseases. Moderna began a phase IIa clinical trial recruiting 600 adult individuals to elucidate antibody response variations and safety with respect to the two dosages of mRNA-1273. On 14 July 2020, the phase I results were released by Moderna, showing that the production of neutralizing antibodies against S1/S2, 15 days’ post-infusion was dose-dependent. Several participants also experienced mild adverse effects such as fever, weariness, migraine, myalgia, and injection site pain in all dose groups. However, these adverse effects were manageable. Based on these results, the dose for the phase III clinical trials was reduced to 200 μg divided into two inoculations with a 29-day gap. An extensive investigational blueprint for the clinical trials by Moderna was announced in September 2020. Moderna announced that if successful, the vaccine could be made accessible to the general public in late March or early April 2021.

The results for Moderna’s lead phase III clinical trial were published recently [23]. The vaccine was approved by the US regulators on 18 December 2020. Analysis of the data shows that the vaccine was 94% successful at preventing symptomatic SARS-CoV-2 disease. The data also suggest that asymptomatic patients may also gain some protection after just one dose of the vaccine. Less severe adverse effects such as headaches were experienced by about 50% of the participants who were vaccinated. Participants in both the vaccinated and the placebo groups rarely experienced serious adverse effects. Severe COVID-19 symptoms were experienced by 30 participants and all of them were in the placebo group.

### 3.2. BioNTech SE and Pfizer Inc., USA

BioNTech and Pfizer announced in December 2020 that they had completed all the primary end-points related to the efficacy of their mRNA vaccine, in a recently concluded phase III human trial. Through a strategic partnership, both companies were successful in developing BNT162b3, which is a nucleoside modified messenger RNA, named modRNA0. This vaccine expresses the vital spike (S) protein found on the protein coat of SARS-CoV-2 to induce immunogenicity against the viral agent post administration. Both the immunogenicity and safety profiles of the agent were established in early-stage clinical trials [26]. The candidate vaccine has already passed through the initial investigational stage of an advanced-stage clinical trial, officially named “A Phase 1/2/3, Placebo-Controlled, Randomized, Observer-Blind, Dose-Finding Study to Evaluate The Safety, Tolerability, Immunogenicity, and Efficacy Of SARS-CoV-2-RNA Vaccine Candidates Against COVID-19 in Healthy Individuals” [27]. The study began on 29 April 2020.

Senior Vice President and Head of Vaccine Research and Development at Pfizer reported that the data obtained from the safety and immunogenicity trials were encouraging and expressed her confidence in BNT162b to serve as the SAR-CoV2 vaccine and save millions of lives. Moreover, Ugur Sahin, M.D., Co-Founder of BioNTech, who is also serving as the chief-executive-officer, stated that collection scientific data of more than 11,000 participants with BNT162b, as well as information from studies conducted previously, exhibit a high breadth of T cell responses and, more importantly, a strong safety profile. These were positive indicators towards the emergence of a safe and effective vaccine candidate. Anticipating the accomplishment of phase III clinical trials, Pfizer and BioNTech decided to manufacture around 100 million dosages before 2020′s end and approximately 1.3 billion doses before the end of 2021 around the world.

The results of the recently concluded phase III trial show that in preventing symptomatic SARS-CoV2 infection, the vaccine proved to be highly efficacious [28]. The trial, which recruited a total of 43,548 participants, assigned them randomly to a placebo group (21,728) and a vaccinated group (21,720). Their analysis of the data shows an efficacy of 95% for the vaccine at preventing symptomatic COVID-19 infections. This analysis was based on 162 COVID-19 cases found in the placebo groups and 8 in the vaccinated group. A similar or higher efficacy was observed when the data were stratified in various age groups. Vaccine efficacy did not decline when they analyzed the data in subgroups based on race, sex, ethnicity, coexisting conditions and baseline body-mass-index. There was only one case of severe COVID-19 infection requiring hospitalization in vaccinated arms and nine in placebo arms. The vaccine was safe, with most of the unwanted effects or reactions reported being mild to moderate. Serious adverse effects were much less common and their prevalence was similar in both vaccinated and placebo groups. These results show that the safety of the vaccine was comparable to other viral vaccines.

### 3.3. Janssen Pharmaceuticals, USA

Another COVID-19 vaccine, using the platform of adenovirus serotype 26 vector (Ad26.COV2-S), was financed by Janssen Pharmaceuticals for the phase 3 clinical trials. This vaccine was a joint effort of the Beth Israel Deaconess Medical Center, with the Janssen’s state-of-the-art AdVac^®^ technology, where a panel of researchers from both Johnson and Johnson (J&J) and Janssen Pharmaceuticals analyzed multiple leads post synthesis. For SARS-CoV-2 infection prevention, Ad26.COV2-S emerged as the lead candidate. On 10 June 2020, J&J reported that in phase 1/2a of the clinical trial, the safety, reactogenicity and immunogenicity of adenovirus serotype 26 (Ad26) was to be studied. On 30 July 2020, the Nature group published data which reported that a robust immune response and the resultant aversion of infection was seen after administering the investigational vaccine. Furthermore, in a pre-clinical study conducted in non-human primates (NHPs), it was found that the vaccine provided protection in the lungs from the virus. A single immunization with Ad26.COV2-S protected against SARS-CoV-2-induced pneumonia, weight loss, and mortality as per a study published in *Nature* on 3 September 2020.

The vaccine contains a non-replicating viral vector vaccine (which uses live infections to convey DNA into human cells), otherwise called JNJ-78436735. The vaccine contains S protein, which is further stabilized by certain modifications [18]. Assessment of the efficacy of the viral vector vaccine is being conducted with regard to the vaccine’s ability to prevent molecularly confirmed moderate to critical/severe COVID infections. The phase 3 study was officially named as,” A Randomized, Double-blind, Placebo-controlled Phase 3 Study to Assess the Efficacy and Safety of Ad26.COV2-S for the Prevention of SARS-CoV-2-mediated COVID-19 in Adults Aged 18 Years and Older”. The study started on 7 September 2020, with approximately 60,000 participants targeted [29]. The results of its phase 3 trial showed that the vaccine is 85% effective in preventing severe COVID-19 infections [30].

### 3.4. Novavax, Inc., USA

In September 2020, a US-based company called Novavax, Inc. initiated the phase III clinical trials of its recombinant spike protein nanoparticle SARS-CoV-2 vaccine (SARS-CoV-2 rS), which is officially called NVX-CoV2373. This interventional study was officially named “A Phase 3, Randomized, Observer-Blinded, Placebo-Controlled Trial to Evaluate the Efficacy and Safety of a SARS-CoV-2 Recombinant Spike Protein Nanoparticle Vaccine (SARS-CoV-2 rS) with Matrix-M1™ Adjuvant in Adult Participants 18–84 Years of Age in the United Kingdom”. Built from the SARS-CoV-2 spike glycoprotein, which performs an integral function in binding of the virus to human ACE2 receptor, NVX-CoV2373 consists of a recombinant SARS-CoV-2 nanoparticle vaccine which is combined with a Matrix-M1 adjuvant for immunogenicity enhancement. The evidence of producing protective immunity in animal models and safety and immunogenicity in humans has already been reported [31,32]. In this most recently conducted trial phase, the effectiveness, safety, and immunogenicity against SARS-CoV-2 infection in roughly 10,000 volunteers aged 18 to 84 years was studied. The investigation planned to enlist a minimum of 25% of individuals more than 65 years old, and likewise focused on populations that are at a higher risk of contracting SARS-CoV-2 disease, including racial and ethnic minorities.

In Australia, the first human safety trials were performed in May 2020. The mechanism of NVXCoV2373 was determined in pre-clinical trials, showing the blockade of the spike protein binding to its receptors (viral molecular targets) through stimulation of antibody production. NVXCoV2373 was reported to be well-tolerated and elicited robust antibody responses in its phase 1 part of its phase 1/2 clinical trial. Phase II clinical trials to assess immunogenicity were conducted in South Africa and Australia, and the trials in the United States of America began in August. Novavax’s president of Research and Development, Gregory Glenn, expected a rapid phase III clinical trial enrollment and to soon provide near-term efficacy results from the investigation. Furthermore, he promised that early results would ease regulatory approval for licensure essential for various nations, such as, but not limited to, the UK, EU and USA. The results of the phase III trial are published recently and report an 89.7% efficacy against COVID-19 infection. The two-dose vaccine was found 86.3% effective against the B.1.1.7 (or alpha) variant and 96.4% effective against non-B.1.1.7 variants [33]. There is significant ease in handling the product, as the formulation is viable for use in an unfrozen liquid state. Moreover, storage conditions at 2–8 °C facilitate relatively easy storage. Novavax hopes to manufacture up to 2 billion annualized doses, once all capacity has been brought online by the end of 2021.

## 4. Vaccine for COVID-19 by Russia

### Gamaleya Research Institute, Russia

Sputnik V, previously called Gam-COVID-Vac, is being produced by the Moscow city government in collaboration with Crocus Medical BV, which is a contact research organization. The sponsors for this endeavor are the Gamaleya Research Institute of Epidemiology and Microbiology and the Health Ministry of the Russian Federation. The vaccine quickly progressed through initial development and entered phase III trials last year. The official name of the phase III trial was “A Clinical Trial of Efficacy, Safety and Immunogenicity of Combined Vector Vaccine Gam-COVID-Vac in SARS-COV-2 Infection Prophylactic Treatment in Republic of Belarus” [34,35].

The study comprised a cohort of 40,000 volunteers divided into two major groups. One group received the vaccine, whereas the other received the placebo. For every participant given a placebo, the vaccine was given to three patients. Therefore, the ratio of vaccinated to placebo-administered individuals was 3 to 1, indicating that 30,000 participants were given the vaccine and 10,000 received the placebo. Moreover, all patients were above the age of 18 and subdivided into five age categories: 18–30, 31–40, 41–50, 51–60, and 60+ years

The study plan comprised six visits per participant for a period of 180 ± 14 days after the first dose of the placebo/vaccine. The first visit was a preliminary checkup by the physician, after which the patient received the vaccine/placebo intramuscularly. The participants received the second dose of the vaccine on the next visit. The next three visits were scheduled for days 28 ± 2, 42 ± 2, and 180 ± 14, during which participants’ health conditions were monitored and recorded in conjunction with any notable adverse effects. To troubleshoot an absence of the patient at the prescribed date, telemedicine was used to ensure that data were recorded and the study continued smoothly.

This vaccine is a combination of two different adenovirus-based vectors and follows a prime-and-boost approach. The two vectors used in this vaccine include a recombinant serotype 26 and serotype 5 adenoviral vectors. A component of the S protein of the novel SARS-CoV-2 is inserted into each of these vectors. Although the preliminary data of the phase 3 trial were not made public, when it was announced on 14 December, Sputnik V was shown to have an efficacy of 91.4% in the trial.

In early November 2020, the Director of the Gamaleya Research Institute of Epidemiology and Microbiology, Alexander Gintsburg, stated that of the 2000 individuals inoculated with both vaccine portions, none had contracted the SARS-CoV2 viral agent. It is important to note, however, that Russia green-lit mass vaccination programs of Sputnik V whilst it was undergoing phase III trials. This has raised the eyebrows of many in the scientific community around the world. Critics argued that safety and efficacy should be evidenced by irrefutable data before taking such a step. Responding to such voices, Mr. Gintsburg defended this strategy by stating that their approach is backed up by credible scientific research.

In addition to Belarus, Sputnik V was also investigated in clinical trials in several other countries, including Venezuela, India and the UAE. Trial investigators also revealed that Sputnik V is even more effective against the most severe cases of SARS-CoV-2, with an efficacy of 100%. The manufacturing and distribution of Sputnik V is being accelerated by the Russian direct investment fund. The demand for the vaccine is huge, as requests for more than 12 billion doses have already been received from about four dozen nations around the world. The Russian direct investment fund is collaborating with manufacturers in South Korea, Brazil, China and India to ramp up the production of the vaccine to fulfill the demand. The vaccine does not require extremely low storage temperature and can be stored at temperatures of +2 to +8 degrees Celsius in the lyophilized form. Sputnik V is one of the cheapest vaccines, costing less than USD 10 for each shot.

## 5. Vaccines for COVID-19 by China

### 5.1. Sinovac Biotech, China

This vaccine, called CoronaVac, is an inactivated virus vaccine manufactured by Sinovac Biotech, a Chinese biotechnology and pharmaceutical company, and was investigated in advanced phase 3 clinical trials in humans last year. CoronaVac is an inactivated or absorbed SARS-CoV-2 vaccine, in which the spike protein is encapsulated in a suitable vector. In the preclinical studies, this vaccine produced strong antibodies specific to the S protein as well as N-specific antibodies [36]. The Butantan Institute was one of the sponsors of this vaccine. The official title of the phase III trial was “A Double-Blind, Randomized, Placebo-Controlled Phase III Clinical Trial to Evaluate Efficacy and Safety in Healthcare Professionals of the Adsorbed COVID-19 (Inactivated) Vaccine Manufactured by Sinovac”. The duration of the phase 3 trial was 3 months, from 21 July 2020 to 21 September 2020 [37]. The vaccine is one of five leads to have entered advanced clinical trial phases from China.

This trial aimed to prevent the individuals given the vaccine from being infected with COVID-19. A total of approximately 8870 individuals took part in the trial. The patients in both the control and experimental arms were equal in number. A placebo was injected into the control group, whereas the remaining patients were given the CoronaVac intramuscularly. Dosing took place in two parts at an interval of 14 days. The primary parameter for determining the efficacy was to detect the number of patients who exhibit symptoms of SARS-CoV-2 in the second week post administration. Safety studies involved the formation of two patient groups. With a follow up period of one year, the adverse effect profile was determined in adults, where adults were considered between the ages of 18 and 59, and elderly patients were those above the age of 60. Adverse effects were considered only if they have a frequency of 1/1000 or 1/500 in the adult and elderly groups, respectively. An interim preliminary analysis to determine vaccine efficacy was released when the study reaches 150 patients.

The vaccine manufacturers targeted a mass vaccination roll out in early 2021. The company aims to supply CoronaVac to the US after completion of the phase III clinical trials; however, obtaining FDA approval may prove to be a challenge. Initially, CoronaVac distribution was mainly China-centric, but since June and July 2020, the focus has shifted to their intention to provide an international supply of the vaccine, especially to the West and Australia.

However, the recently reported results of CorornaVac showed a lower efficacy in a phase 3 clinical trial in Brazil compared with over 90% efficacy rates for several other recently approved vaccines. The efficacy rate of CoronaVac was lower than the results obtained in the phase 3 trials of the same vaccine in Indonesia and Turkey. The Butantan Institute in São Paulo, which carried out the trial, initially reported the efficacy of the vaccine to be 78%. However, it was found that when the criteria were narrowed down to include people who required medical attention, the figure dropped to barely above 50%.

However, the investigators suggested that it would still be helpful in countries which are facing difficulties in stemming the pandemic and where outbreaks of the virus are wreaking havoc. They indicated that although the efficacy of the vaccine is low, there were no hospitalizations with severe symptoms of the disease among the 252 recorded cases in the Brazilian investigation. Among the 9200 healthcare workers who participated in the study, 85 cases in the vaccinated group were reported, while among those who received the placebo, the number of reported cases was 167. In a separate study of the same vaccine conducted in Turkey, the results of 1322 volunteers showed an efficacy rate of 91.25%, as there were only 29 COVID-19 cases reported.

A similar trial of the same vaccine conducted in Indonesia exhibited an efficacy of 65.3% at preventing symptomatic COVID-19 infections. In the trial in which 1600 participants were involved, only 25 COVID-19 cases were reported. On the basis of these results, Indonesia issued emergency use authorization for CoronaVac and started its national vaccination drive with the vaccine from 13 January 2021. Even at 65% efficacy rate, the number of people who might benefit from this vaccine, given the size of the Indonesian population, could be huge. In the Brazilian trial, no hospitalizations suggests that this vaccine would be successful in preventing severe cases of the disease.

### 5.2. Wuhan Institute of Biological Products/Sinopharm, China

WIBP-CorV, which is the product of a strategic partnership between the Wuhan Institute of Biological Products, LTD, and Sinopharm, is approved in many countries, where it has been given emergency use authorization for vaccinating their populations against COVID-19 infection. Several countries, some of which have completed their interim analyses, are conducting phase III clinical trials for the vaccine. The inactivated novel SARS-CoV-2, or nCov, is the main component of the formulation. The official name of their first phase III trial was, “A Randomized, Double Blind, Parallel Placebo Controlled, Phase III Clinical Trial to Evaluate the Safety and Protective Efficacy of Inactivated SARS-CoV-2 Vaccine in Healthy Population Aged 18 Years and Above”. This trial was conducted in the United Arab Emirates and was approved by the Abu Dhabi Health COVID-19 Research Ethics Committee [38].

Healthy patients were recruited for the study to determine the effectiveness of the lead alongside its safety. The trial was similar to many others already discussed. The placebo was given to the respective group in two shots and the vaccine candidate was also administered twice within the space of 21 days. The number of patients recruited in this study was 40,000, the same as the Sputnik V trial mentioned above. The objective was to find out the extent to which the vaccine provides protection, for at least 2 weeks, against COVID-19 infection.

It is safe to say that China has been leading the race alongside the US with regard to the development of a SARS-CoV-2 vaccine that can readily be available to the public. A practice similar to that of Russia’s Sputnik V has been observed in China as well, where announcements that several Chinese citizens have been immunized despite the fact that phase III trials are still ongoing have been made. However, the major difference is that the individuals were vaccinated under the government-authorized, emergency use program. Chinese regulations do allow such practices; however, the scope and timing are reserved for health emergencies. It was additionally reported by the Chinese government authorities that high-risk people will be prioritized, suggesting frontline medical professionals and senior citizens were the main groups to get the vaccines initially.

Although emergency use authorization in high-risk individuals was already granted, the conditional approval for the general public was approved by the Chinese government in late December 2020, after it was revealed that the efficacy rate of the vaccine was 79% in phase III clinical trials. The phase 3 clinical data of the trial was published recently [39]. The same vaccine has already been approved by the governments of Bahrain and UAE for vaccinating high-risk individuals.

The Chinese government asserts that the approval was granted only after all the pre-set requirements for such approval were complete. They reiterated that all the clinical data were critically analyzed, required verifications and tests done and strict reviews were carried out before making final approvals. They emphasized that it is only a conditional approval at the moment. This approval is likely to enable the Chinese government to vaccinate high-risk groups including the elderly, healthcare workers and other more vulnerable groups. Several million doses of the vaccine have already been injected, but the government hopes that several million more vaccinations will be carried out soon. However, the scientific community is hesitant to comment on the efficacy of the vaccine or the Chinese government’s vaccination program with this vaccine, as the data are not yet published or peer-reviewed. The scientific community cannot simply evaluate the vaccine on the basis of press releases and statements by government officials. However, more data are likely to emerge as vaccinations begin in other countries.

### 5.3. CanSino Biological Inc./Beijing Institute of Biotechnology, China

An Ad5-nCov-containing vaccine created by the collaboration between Beijing Institute of Biotechnology and CanSino Biological Inc was investigated in healthy individuals above the age of 18. The vaccine was studied in an advanced clinical trial, known officially as “A Global Multicenter, Randomized, Double-blind, Placebo -Controlled, Adaptive Designed Phase Ⅲ Clinical Trial to Evaluate the Efficacy, Safety and Immunogenicity of Ad5-nCoV in Adults 18 Years of Age and Older”. The goal of the study was to determine the immunogenicity as well as the safety and effectiveness of the lead. The trial commenced on 15 September 2020. The investigation recruited approximately 40,000 individuals, of which 20,000 were in the experimental cohort, and others were in the placebo arm of the examination. This vaccines is given intramuscularly as a single shot [40]. The Aga Khan University hospital Karachi, Shaukat Khanum Memorial Cancer Hospital and Research Center, Lahore, and Shifa International Hospital, Islamabad were the centers for recruiting the volunteers to conduct the trial.

The ability of this vaccine to prevent COVID-19 disease was the baseline parameter to determine the efficacy. Therefore, the primary outcome measures of the phase III trial include prevention of COVID-19 cases post vaccination from day 28 to 12 months. The incidence of drastic undesired effects was measured in a timeframe of 12 months. Other results measured the incorporated frequency of serious COVID-19 cases from day 14 to a year post immunization. The frequency of undesired effects between day 0 and day 7 post immunization was estimated, as well as the incidence of unsolicited adverse reaction between day 0 and day 28 post vaccination. The immunogenicity of S-RBD IgG immunoglobulin through the ELISA technique was also determined 28 days post injection. The seroconversion of S-RBD IgG immunoglobulin after immunization and the cell-mediated immunity reaction profile from day 28 post immunization were also measured as secondary end-points.

### 5.4. Beijing Institute of Biological Products/Sinopharm, China

A multi-company venture of the Huesped Foundation, China National Biotec Group Company Limited, and Beijing Institute of Biological Products Co., Ltd., and sponsored by the Laboratorio Elea Phoenix S.A., created a vaccine for which the phase III clinical trial was conducted in an Argentinian population. The trial investigated an inactivated viral vaccine. In such vaccines, the viral pathogen is inactivated by simple heating or by chemical means. The virus subsequently fails to produce disease. However, it is able to maintain its antigenic characteristics, which facilitates an adaptive immune response in the host. Also called BBIBP-CorV, the formulation constituents comprise SARS-CoV-2 which is inactivated. The virus is extracted from renal cells of the African Green monkey infected with strain HB02 of the SARS-CoV-2 virus. This vaccine was shown to produce a strong antibody response in non-human primates in the pre-clinical studies. Volunteers who were administered this vaccine are expected to produce an immune response against SARS-COV-2. This vaccine has already shown in the phase I/II trials that it can produce high levels of antibodies against SARS-COV-2 and is safe.

Its phase III trial is officially entitled “A Randomized, Double Blind, Placebo Parallel-controlled Phase III Clinical Trial to Evaluate the Efficacy, Immunogenicity and Safety of the Inactivated SARS-COV-2 Vaccine (BBIBP-CorV) in Argentine Healthy Population Aged Between 18 and 85 Years” [41]. The study started recruiting volunteers on 16 September 2020, and approximately 3000 volunteers have been recruited in a randomized control trial in which the interventional model is a parallel assignment of candidate vaccine and placebo to two separate groups of volunteers. The investigation is double-blinded in which both the physician and the patient do not know if they are part of the vaccine or placebo group.

## 6. Vaccine for COVID-19 by India

### Bharat Biotech International Limited, India

The BBV152 vaccine was developed by Bharat Biotech to stop the further spread of the SARS-CoV-2 pandemic, which has adversely affected the Indian population. A phase III clinical trials commenced last year. The primary objective of this investigation was to find out the effectiveness of BBV152 at preventing symptomatic COVID-19 infection. This was done by evaluating the efficacy, safety, and immunogenicity of the vaccine by following up the participants up to 1 year after the administration of the second dose [42]. The sponsors of this study are Bharat Biotech International Limited, in collaboration with the Indian Council of Medical Research and Iqvia Pty Ltd. The recently published phase I trial of this vaccine suggests that BBV152 can enhance immune responses and possesses a tolerable safety profile [43].

In the phase III clinical trial, the participants were assigned in a randomized manner to the two groups (vaccine and placebo groups) in a double-blind study in which several hospitals took part across India. The trial was an event-driven investigation. The immunogenicity, safety and efficacy were explored in participants age 18 and above. The ratio of the participants who received the experimental vaccine and those who received the placebo was 1:1. The total number of participants that were enrolled in the study was over 25,000. A nasopharyngeal swab was collected from all participants before the first dose to properly assess the effectiveness and safety of the vaccine. A positive reverse transcriptase polymerase chain reaction test or anti-SARS-CoV-2 IgG antibody test did not affect the recruitment of the participants. However, their data were not part of the primary efficacy calculations. Safety check-ups for all individuals were conducted as per established protocols.

The investigational sites were divided into three categories. In the first category, symptomatic patients were followed up and their blood and nasal swabs taken every 15 days for the detection of COVID-19 infection. In the second category, samples were collected from asymptomatic individuals as well at an interval of 1 month. In the third category, samples were collected for carrying out immunological assessments in addition to the detection of COVID-19 infections.

## 7. Vaccine for COVID-19 by Canada

### Medicago, Inc., Canada

The Medicago vaccine, called coronavirus-like particle (CoVLP), uses a plant-based platform. Their approach produces non-infectious virions using living plants as bioreactors. In order to establish the safety, immunogenicity and efficacy of their vaccine, a phase II/III trial was started to optimize the dosing regimen and chosen formulation for CoVLP. The trial, in which participants were tracked for up to 365 days after the final dose of the vaccine for safety, immunogenicity and efficacy purposes, was an event driven study. The trial compared the CoVLP formulation with the placebo in participants randomly assigned to the two groups [44].

The number of volunteers in the trial was over 30,000, and these volunteers were randomized in the vaccine or placebo group. The official title of the of the investigation was “Randomized, Observer-Blind, Placebo-Controlled, Phase 2/3 Study to Assess the Safety, Efficacy, and Immunogenicity of a Recombinant Coronavirus-Like Particle COVID-19 Vaccine in Adults 18 Years of Age or Older.” The experimental arm cohort of the study was given 3.75 µg of CoVLP with an adjuvant AS03 (0.5 mL) in the deltoid region of the arm, with each participant receiving two doses, 21 days part, in alternating arms. Participants in the placebo group received two doses of placebo (0.5 mL) in alternating arms, 21 days apart. This study started in November 2020. The company say that CoVLP vaccine would be the first one derived from plants to reach the market for human use. Medicago, Inc., is the only company capable of producing this vaccine at the commercial scale [45]. The company seem to have the ability and the capacity to manufacture millions of doses at their facilities in Canada and the USA.

## 8. COVID-19 Vaccines and the Variants of Concern

SARS-CoV-2, which comprises a protein coat and positive sense single stranded RNA, is subject to mutations. It falls under Class IV of the Baltimore classification of viral particles. Similar to other viruses, most mutations are nonsense mutations, while some prove to be deleterious to the virus. However, in response to certain selection pressures, mutations can arise that increase the virulence of the virus, either by enhancing the disease-causing capacity of the viral agent or by increasing the robustness of the virus against host defense mechanism. Additionally, it is important to note that these variants have a stronger transmissibility profile, and soon these variants will be detected in large-scale populations [46].

The WHO’s COVID-19 Reference Laboratory Network [47] has been tracking SARS-CoV-2 genomic mutations since the pandemic’s start. As the pandemic progressed, variants of the SARS-CoV-2 emerged throughout the world. The WHO’s Virus Evolution Working Group was setup in June 2020 to search and analyze these variants, their phenotype and how they will likely influence the countermeasures taken. Furthermore, a global risk-monitoring framework for coordinating various international system components to monitor as well as to assess the SARS-CoV-2 variants and their impact was developed by the WHO. This was set up to gather, investigate and share information to recognize urgent priorities, improve the limitations of diagnostic laboratories, technical networks and expert groups. The WHO has given working meanings of variations of interest (VOIs) and variations of concern (VOCs) that will be refreshed when required [48]. To study the ‘variants of interest’—which are SARS-CoV-2 strains that lead to increased infections locally—the use of the Greek alphabet has been recommended by an expert committee of the WHO, and the same nomenclature is suggested for the more dangerous SARS-CoV-2 mutants, also referred to as the ‘variants of concern’ [49].

The long-awaited system is intended for use by the media, policymakers and the public, and is published recently [46]. It should have come earlier, because its absence has fueled the practice of naming variants after the places in which they were discovered, such as the ‘Kent variant’, which is otherwise known as B.1.1.7. Under the WHO’s new system, B.1.1.7 is also called Alpha, B.1.351 is Beta, P.1 is Gamma. The B.1.617.2 lineage, first identified in India, is now called Delta [50].

Despite the rapid global licensure and roll out of vaccines in various countries, the number of individuals vaccinated still represents a small fraction of the global population [51]. To better understand the effect of genomic variations on the effectiveness of vaccines, both orthodox viruses and pseudo-viruses possessing particular spike mutations (either individually or in combination) and larger sets of mutations representing variants of concern and other circulating spike mutations have been assessed by neutralization assays with postvaccination sera [51]. On average, studies report a fold change in variant virus, or pseudo-virus neutralization relative to wild-type virus (the serum concentration at which 50% neutralization (IC_50_) is achieved with the variant divided by the average IC_50_ for the wild-type virus).

### 8.1. Laboratory Studies

High binding titers for IgM and IgG anti-SARS-CoV-2 spike immunoglobulins with plasma neutralizing activity and RBD-specific antibodies equivalent to those in natural infection were observed in 20 volunteers immunized with the mRNA-1273 vaccine (Moderna) or BNT162b2 (Pfizer–BioNTech) after vaccination [52]. Additionally, epitope mapping results indicated that mAbs extracted from individuals immunized exhibited targeting of a range of RBD epitopes similar to those isolated from naturally infected individuals [52]. Interestingly, the numbers of memory B-cells specific to RBD and plasma neutralizing activity of the post-vaccination sera immunized participants were found to be equivalent to those of plasma from individuals who had recovered from natural SARS-CoV-2 infection [52]. Studies using pseudoviruses containing RBD-mutations carried by variants of concern indicated that within the vaccinated individuals, the neutralizing activity of plasma demonstrated a significant decrease of onefold to threefold against E484K, N501Y or the K417N + E484K + N501Y triple mutant [52]. The effect of N501Y alone on neutralization is relatively modest, and other research using sera from 20 participants in a trial of the BNT162b2 vaccine showed neutralizing titers equivalent to those of pseudoviruses possessing the N501 and Y501 mutations [53]. Other investigations with recombinant viruses carrying N501Y, ΔH69–V70 + N501Y + D614G or E484K + N501Y + D614G showed that only E484K + N501Y + D614G were responsible for a small and modest reduction in neutralization elicited by two BNT162b2 doses [54].

### 8.2. Clinical Studies

Building upon the neutralization assay, the efficacy of the developed vaccines against variants has been exhibited in multiple clinical investigations. Preliminary reports show little deviation from the observations made in laboratory studies, where the B.1.351 variant has shown the greatest potential for vaccine resistance. For instance, with regard to the B.1.351 variant, the ChAdOx1 nCoV-19 vaccine was unable to provide protection against mild to moderate disease, and the efficacy of the vaccine against the variant was estimated at 10.4% [55,56,57]. However, the same vaccine was successful in inducing immunity against the B.1.1.7 variant. Furthermore, the same is the case for the NVX-CoV2373 (Novavax) protein-based vaccine, which has demonstrated efficacies of 95.6%, 85.6% and 60% against the wild-type, B.1.1.7 and B.1.351 SARS-CoV-2 strains. Moreover, while 72% protection against moderate to severe SARS-CoV-2 infections was observed in the USA, a drastic decrease to 57% in South Africa (at a time when the B.1.351 variant was widespread was also observed with the single-dose vaccine formulated by Johnson & Johnson/Janssen: JNJ-78436735 [58]. From these clinical studies, it is reasonable to conclude that there is significant variation in vaccine efficacy with regard to different SARS-CoV-2 viral strains.

Not only vaccines, but the effect of various therapeutic mono-clonal antibodies (mAb) was investigated on the mutations within SARS-CoV-2 [51]. Single mAb treatment, by serving as a selection, can induce mutations that accentuate antigenic escape. This problem can be solved by the two or more mAbs targeting non-overlapping epitopes in the form of cocktails. REGN-COV2 (Regeneron) (included in the RECOVERY trial in the UK) and AZD7742 (AstraZeneca) are two examples of mAbs cocktails that have been developed [59]. Importantly, some mutations in the RBM have already been identified in variants which are circulating in the UK (for example, N439K, T478I and V483I) and are likely to impact antigenicity.

## 9. Vaccine Effectiveness

Several hundred million people have been vaccinated against the COVID-19 in the last six months. In fact, about 1.7 billion doses of the various COVID-19 vaccines have been administered so far. Now, researchers are analyzing the data regarding the effectiveness of these vaccines. Several of these vaccines showed more than 90% efficacy in the phase III clinical trials. One of the first studies on the Pfizer vaccine to test its effectiveness outside clinical trials, which can exclude some unhealthy individuals or those taking medicines that suppress immune responses, showed 64% effectiveness in long-term-care residents with a median age of 84. In health-care workers, its effectiveness was 90% [60].

A nationwide vaccination campaign in Israel found the Pfizer–BioNTech vaccine to be 95% effective against SARS-CoV-2 infection seven days or more after the second dose [61]. Recently, the effectiveness of the Moderna vaccine is also shown to be over 89% [62]. The Gamaleya National Research Center of Epidemiology and Microbiology in Moscow and the Russian Direct Investment Fund announced that their Sputnik V vaccine has been 97% effective in almost 4 million people in Russia [63].

## 10. Vaccines and Adverse Effects

In April 2021, after a possible link was found between rare blood clots and the COVID-19 vaccine developed by Oxford–AstraZeneca [64,65], the European Medicine Agency (EMA) recommended caution [66]. A week later, the administration of the vaccine developed by the Johnson & Johnson (J&J) of New Brunswick, New Jersey, was recommended to be temporarily stopped because of the suspected cases of unusual blood clots, by the US regulators [67], although the number of suspected cases was only six out of seven million doses of the vaccine administered up until then. In the wake of this decision, J&J has temporarily suspended the distribution of their vaccine to some countries.

The impact of these decisions was felt far and wide. As a result, many countries have decided to restrict the use of the AstraZeneca vaccine to certain age groups, slowing the administration of the vaccine, and some countries, such as Denmark, have stopped using this vaccine completely. All this is fueling further confusion, despite regulators and researchers unequivocally stating that the benefits of the vaccine far outweigh the risk it poses. One reason for this level of confusion is the incomplete, raw and capricious real time data available to everyone around the world.

Scientists are still investigating the link between the use of these vaccines and the rare blood clotting disorders. These blood clots have some common features, including their occurrence in unusual parts of the body such as in the abdomen or the brain. They are also coupled with low levels of blood platelets. Several of the features of these blood clots are common with a rare blood disorders in patients taking heparin, called heparin-induced thrombocytopaenia (HIT) [68,69,70]. However, those who received the vaccine had not taken this drug. Despite these findings, EMA has made it clear that the protection afforded by the vaccines clearly outweighs the risks. The vaccine has demonstrated without any doubt that it prevents against COVID-19 and reduces hospitalizations and deaths [66]. Recently, several national governments have restarted vaccinating their population with the AstraZeneca and J&J vaccines.

## 11. Vaccine Manufacturing Challenges

As soon as COVID-19 vaccines received emergency use authorization, several hundred million doses of the vaccines were manufactured by the pharmaceutical countries in a few months. However, many more vaccine doses are required and fast. In fact, billions of vaccine doses are needed to stem the epidemic [71]. Pharmaceutical companies believe that they will be able to produce enough vaccines by the end of 2021 to vaccinate the entire world’s population. However, this has been complicated by a myriad of political decisions, including imposing questionable export controls and selling most of the vaccine doses to richer countries. One good outcome of this unfortunate situation is that now there is a serious discussion going on to temporarily withdraw intellectual property rights so that vaccines could be made by those poorer countries who have the necessary infrastructure for vaccine production.

The world’s largest manufacturer of vaccines, the Serum Institute of India (SII) in Pune, was expected to produce 100 million doses per month and to contribute significantly to COVAX. However, after a fire broke out at a facility in January, its production capacity was severely affected, currently standing at roughly 60 million doses per month [72]. The SII was given the license to manufacture one billion doses of Covishield by AstraZeneca last June, to fulfill the vaccine requirements of low and middle-income countries. However, export restrictions by the Indian government have resulted in halting the exports only after 64 million doses. COVAX received only 28 million of those doses. The chief executive of the SII defended himself by saying that the Indian government has directed them to prioritize Indian needs. According to Gavi, which leads COVAX with WHO, the surge in Indian COVID-19 cases could delay planned deliveries to 64 lower income countries through COVAX.

The situation of vaccine manufacturing in the Africa is even worse. A continent of over 1.2 billion people, residing in 54 countries, imports 99% of its vaccines and manufactures only 1% of the vaccines it administers [73]. Additionally, as rich countries are favored, Africa finds itself at the back of the queue. It seems that people living in the poorest countries of the world will have to wait till the end of 2022 or 2023 to get vaccinated [71].

## 12. Global Distribution of COVID-19 Vaccines

With the development of vaccines by Moderna, Pfizer, AstraZeneca, and Sputnik V, which boast efficacies of 94.1%, 95%, 82%, and 91.6%, respectively [28,74,75], there is high hope that normalcy will return soon. Additionally, a significant number of vaccines are undergoing phase 3 clinical trials [76]. A two-dose regimen with a three week gap is recommended for these candidates [77]. However, it must be kept in mind that a significant reduction in transmission and mortality is contingent upon the fact that a significant percentage of the global population is immunized. Therefore, it can be safely expected that a shortage of vaccines may occur during the early months of their availability. Even first-world countries such as the US, Australia and those of the EU, which have stockpiled the greatest number of vaccines, will suffer from a shortage initially. However, in the second- and third-world nations, also called low- and middle-income countries (LMIC), the situation could be far worse due to the fact that vaccine supplies will reach them later and in lesser quantities. The limited vaccine supply for LMIC could prove to be severely detrimental for the overall health and economy of those nations, which will also put the global population at risk [78].

For most of the vaccines currently available to the public, the immunization scheme revolves around the deployment of dual dosages [79], but the logistics of a two-dose vaccination campaign, where individuals who have received the first vaccine jab also receive the second one, are challenging, with a special emphasis on limited vaccine supply and the fact that the vaccines have a low shelf-life [80]. Yet, there are methods to circumvent this problem. The fractional dosing technique has been employed to control outbreaks of other contagions to great effect. In this technique, people receive a quantity lower than the recommended dosage of vaccine, which has proven, successfully, to be a way to stretch vaccine supply. Another mechanism that has been implemented to great effect involves a single-dose campaign. For example, to fight against the cholera outbreak in the African nation Zambia, the population was injected with one shot of the dual-dose killed oral cholera vaccine and, months later, high-risk individuals were offered a second dose [81]. Similarly, in 2016, with regard to the yellow fever outbreak in the Democratic Republic of Congo, Uganda and Angola, the populace was administered one fifth of the recommended dosage of the yellow fever vaccine [82]. This vaccination campaign strategy proved to be successful in curbing the yellow fever epidemic in these countries. For multiple reasons, such as the ease of logistical implementation, cost effectiveness, and rapid immunization of the global population, this would be sufficient to induce herd immunity that will in turn allow key community activities, such as the opening of markets, schools and restaurants. Therefore, a single-dose immunization campaign for COVID-19 is an attractive strategy [83,84,85]. However, for this campaign to be realized, the vaccines should not only mitigate disease but also effectively stop infection and hence transmissions to other individuals. Some of these questions are yet to be answered and data to answer such questions are still lacking [23,28,86]. Alongside the aforementioned hurdle, the single COVID-19 vaccine dose campaign is contingent upon the extent of protection that the dose can provide. However, an intrinsic trade off with regard to this strategy is that in an attempt to vaccinate a large chunk of the populace in a small period of time, the durability of the induced immunity may be compromised.

## 13. Vaccine Passports

As the world in being vaccinated and lockdowns are being eased, travel is increasing. However, travel to other countries still poses another challenge. One has to prove that one is vaccinated with the ‘right’ vaccine, because different countries have certified different vaccines for allowing travel to those countries. This vaccine passport is likely to create further inequity and confusion. For example, vaccine passports could further privilege citizens, including researchers based in high-income countries, who already dominate the field of global health. People living in low- and middle-income countries, especially researchers, are already facing problems travelling internationally to attend workshops, conferences and seminars [87]. Furthermore, unequal vaccine allocation and distribution ensures that low- and middle-income countries will take a long time to fully vaccinate their population. Therefore, this will aggravate the feelings of exclusion and lack of diversity around the world.

World leaders and people who stand for fairness and care about global health should actively promote the equal allocation and distribution of vaccines [88]. With the advent of vaccine passports, the already existing divide between the rich and poor countries for vaccine access is going to be exacerbated and may permeate other aspects of global health. In particular, if vaccine passports become necessary for international travel, we must ensure that everyone in the world is vaccinated as quickly as possible.

## 14. Concluding Remarks

It takes a long time to develop a vaccine. There are multiple reasons for this. The initial step is to determine the vaccine’s efficacy and safety in animals. This requires around a half year of rigorous animal care, following guidelines and laboratory regulations, and finishing preclinical investigations. When its safety and efficacy in animals is established, at that point the lead candidate is moved further into human clinical trials. Industrial production is required for extensive clinical trials. Although stage I and II of the clinical trials are modest, stage III is a much bigger phase requiring a huge cohort in each investigational setting. Whether the lead is safe or not is assessed in stage I trials, while stage II is reserved for determining efficacy. The efficacy and safety are assessed on a much bigger scale in stage III clinical trials, after which the regulatory bodies decide whether to approve or reject the vaccine candidate. Although the hastening of this whole cycle might be attributable to the seriousness of the COVID-19 pandemic, efficacy and safety should not, and were not, undermined at any stage. Even after guidelines are changed to speed up the vaccine production, it was still unrealistic to anticipate the development of safe and efficacious vaccine inside a half year after the beginning of clinical trials. Further difficulties might be confronted after a vaccine is approved for general use, particularly manufacturing at the scale to vaccinate the entire world population. This would be more probable if increasing the production includes new technologies that have not been tested beforehand.

Important challenges that lie ahead, once the vaccines are approved for the use in general public, are myriad and include the optimization of doses, boosters and their schedules which, due to the urgency of vaccine development, pharmaceutical companies might not have explored in detail [89]. The strategy of fractional dosing, previously used for extending the supplies of yellow fever vaccine, can be employed here [90,91]. A higher efficacy is reported by the AstraZeneca vaccine for a regimen where a full second dose is administered to the individual after they have receiving a half first dose [17]. For the Sinovac vaccine, a 4-week dosing interval, which is greater than the previously practiced 2-week interval, could possibly increase the concentration of antibodies [92]. These strategies might help in extending the supplies of COVID-19 vaccines for global coverage.

The fight against the SARS-CoV-2 pandemic is still not finished, and the development of safe and efficacious vaccines as well as their manufacturing at scale, are the need of the hour. Although the speed at which vaccines are currently being manufactured all across the globe shows the seriousness of this endeavor, the fact that some vaccines could not prevent infection in humans despite showing promising results in pre-clinical studies, and that others have been shown to be associated with lung damage and other severe complications are all warning signs that there is little to no room for error. To ensure that the fallout is minimal, extensive long-term studies that successfully establish that the vaccines are safe are vital. Furthermore, post immunization, the individual should have long-lasting immunity against SARS-CoV-2. This is crucial because SARS-CoV-2 has shown to have a relatively high reinfection potential, where individuals suffer from symptoms after contracting the pathogen again, albeit at a much milder magnitude. Therefore, immunity offered by the vaccines should last for many months and preferably for years. Lastly, the vaccine should offer prolonged protection to high-risk population demographics such as the elderly, whose immune systems are weaker as compared to the younger population. We should ensure that the ideal vaccine must provide protection to this most vulnerable section of our population.

## 15. Expert Opinion

We may not see the end of COVID-19 pandemic without the global roll-out of safe and effective vaccines that produce enough herd immunity. Several countries around the world have authorized emergency use of various vaccines and mass vaccination drives have already ensued in these countries. However, several challenges remain to be addressed to achieve global control of COVID-19, including meeting the global vaccine demand, their affordability, and their wide deployment in the communities.

As mentioned earlier, one major challenge in the short-term would be meeting the high global demand for different vaccines. However, this challenge will be addressed as pharmaceutical countries bring more and more production capacity online. Towards the end of 2021, we might see a vaccine glut. In fact, the greater challenge may be to address the vaccine hesitancy extensively prevalent not only in developing countries but also in technologically advanced countries with a high literacy rate. Herd immunity is not possible without a significant portion of the population developing neutralizing antibodies.

Another challenge will be the emergence of new viral strains that may be resistant to the currently employed vaccines. However, with so many vaccines in clinical use and even more under development, and with so many different platforms utilized for the development of these vaccines, there is high likelihood that any novel viral variant will respond to at least a few vaccines.

Vaccine nationalism may also affect the availability of the vaccines to poorer countries. This is evident from the reports which suggest that billions of doses of vaccines from AstraZeneca, Pfizer and Moderna have already been procured by the countries from Western Europe and the USA. Some of these countries have ordered vaccines shots many times their population sizes. This will likely prevent several hundred million people from poorer countries from getting vaccinated. COVID-19 Vaccines Global Access (COVAX) led by UNICEF, and similar vaccine alliances, are doing their best to ensure equitable access to COVID-19 vaccines.

One important area of concern is the low number of elderly volunteers recruited in most of the vaccine trials, which makes it challenging to generalize the clinical trial findings to people over the age of 60. There is an urgent need to enlist more elderly volunteers in the ongoing and future clinical trials. This will help us to gain more knowledge about the development of immunity against COVID-19 in the elderly and whether this immunity is enough to protect them from the infection and for how long. Another important group missing from the clinical trials is children. Although COVID-19 has largely spared children, as observed in the last year, it is still possible that the newer strains affect children more. Therefore, it is important to include more children in the trials to accumulate more data about the vaccine protection afforded to children and any other effects they may or may not produce. It is encouraging to see that the Pfizer vaccine is recommended for children between 12 and 18 after the data from the clinical trials suggested that it is safe and effective in this age group. These findings will help us in recommending guidelines for the use of vaccines in protecting children and the elderly, the two most vulnerable segments of our society, from this deadly pandemic.

## Figures and Tables

**Table 1:** Most promising vaccines, their developers, platforms and constructs, and the clinical phases in which these vaccines are currently being tested [2].

Developer	Platform	Construct	Development Phase	Vaccine
University of Oxford/AstraZeneca, UK	Virus vector (ChAdOx1)	Full length S	Phase III	AZD1222
Moderna/NIAID, USA	LNP- mRNA	Full- length S with two proline substitutions	Phase III	mRNA-1273
BioNTech SE and Pfizer Inc. USA	LNP- mRNA	Full- length S with two proline substitutions	Phase III	BNT162b2
Gamaleya Research Institute, Russia	Virus vector (Ad26 and Ad5)	Full length S	Phase III	Sputnik V
Sinovac Biotech, China	Inactivated virus	NA	Phase III	CoronaVac
Wuhan Institute of Biological Products/Sinopharm, China	Inactivated virus	NA	Phase III	WIBP-CorV
CanSino Biological Inc./Beijing Institute of Biotechnology, China	Virus vector (Ad5)	Full length S	Phase III	Ad5-nCoV
Janssen Pharmaceuticals, USA	Virus- vectored (Ad26)	Full-length S with two proline substitutions and 3 mutations at furin cleavage site	Phase III	Ad26.COV2.S
Novavax, USA	Protein subunit (CHO)	Full-length S with two proline substitutions and 3 mutations at furin cleavage site	Phase III	NVX-CoV2373
Beijing Institute of Biological Products/Sinopharm, China	Inactivated virus	NA	Phase III	BBIBP-CorV
Bharat Biotech BBV152 vaccine	Inactivated virus	NA	Phase III	BBV152
Medicago Vaccine	Virus like particle	NA	Phase III	Coronavirus like particle

**Table 2:** Summary of the platforms, dosing schedule, no. of doses and route of administration of candidate vaccines in the clinical trials [2].

Platforms of the Candidate Vaccines			
Platform		Candidate Vaccine (No. and %)	
PS	Protein subunit	20	31%
VVnr	Viral Vector (non-replicating)	10	16%
DNA	DNA	8	13%
IV	Inactivated Virus	9	14%
RNA	RNA	7	11%
VVr	Viral Vector (replicating)	4	6%
VLP	Virus Like Particle	2	3%
VVr + APC	VVr + Antigen Presenting Cell	2	3%
LAV	Live Attenuated Virus	1	2%
VVnr + APC	VVnr + Antigen Presenting Cell	1	2%
**Dosing Schedule and No. of Doses of Candidate Vaccines**			
**No. of Doses**	**Dosing Schedule**	**Candidate Vaccine (No. and %)**	
1 dose	Day 0	12	19%
2 doses	Day 0,14 (5), Day 0, 21 (16), Day 0, 28 (17)	38	59%
3 doses	Day 0, 28, 56	1	2%
No Data	NA	13	20%
**Route of Administration of Candidate Vaccines**			
**Route of Administration**		**Candidate Vaccine (No. and %)**	
PO	Oral	3	5%
SC	Subcutaneous	2	3%
ID	Intradermal	3	5%
IM	Intramuscular	48	75%
	No Data	8	13%

## Data Availability

Data are contained within the article or supplementary material.
